# Lipophilic Toxins in Galicia (NW Spain) between 2014 and 2017: Incidence on the Main Molluscan Species and Analysis of the Monitoring Efficiency

**DOI:** 10.3390/toxins11100612

**Published:** 2019-10-22

**Authors:** Juan Blanco, Fabiola Arévalo, Jorge Correa, Ángeles Moroño

**Affiliations:** 1 Centro de Investigacións Mariñas (CIMA), Consellería do Mar. Xunta de Galicia. Pedras de Corón s/n, 36620 Vilanova de Arousa, Spain; 2 Centro Tecnolóxico para o Control do Medio Mariño de Galicia (INTECMAR), Consellería do Mar. Xunta de Galicia. Peirao de Vilaxoán s/n, 36611 Vilagarcía de Arousa, Spain; farevalo@intecmar.gal (F.A.); amoronho@intecmar.gal (Á.M.); jcorrea@intecmar.gal (J.C.)

**Keywords:** lipophilic toxins, bivalves, Galicia, spatial variability, temporal variability, biotransformation, monitoring, risk

## Abstract

Galicia is an area with a strong mussel aquaculture industry in addition to other important bivalve mollusc fisheries. Between 2014 and 2017, 18,862 samples were analyzed for EU regulated marine lipophilic toxins. Okadaic acid (OA) was the most prevalent toxin and the only single toxin that produced harvesting closures. Toxin concentrations in raft mussels were generally higher than those recorded in other bivalves, justifying the use of this species as an indicator. The Rías of Pontevedra and Muros were the ones most affected by OA and DTX2 and the Ría of Ares by YTXs. In general, the outer areas of the Rías were more affected by OA and DTX2 than the inner ones. The OA level reached a maximum in spring, while DTX2 was almost entirely restricted to the fall–winter season. YTXs peaked in August–September. The toxins of the OA group were nearly completely esterified in all the bivalves studied except mussels and queen scallops. Risk of intoxication with the current monitoring system is low. In less than 2% of cases did the first detection of OA in an area exceed the regulatory limit. In no case, could any effect on humans be expected. The apparent intoxication and depuration rates were similar and directly related, suggesting that the rates are regulated mainly by oceanographic characteristics.

## 1. Introduction

Some phytoplankton species produce substances that are toxic to humans [1,2]. These species can bloom during certain periods of the annual cycle of phytoplankton and may be ingested by bivalve molluscs (cultured or in natural beds) which retain and ingest most of the particles suspended in the water, including toxin-containing cells [3,4,5,6,7,8,9]. Once ingested, a proportion of the toxins is absorbed by the bivalves, which frequently accumulate it to levels that are toxic to humans [3,10]. In such cases, the risk to human health from consuming bivalves is high and therefore, the harvesting and marketing of these animals must be prohibited, leading to losses for both the fisheries and aquaculture sectors [11]. Many countries run monitoring programs for marine biotoxins to minimize this risk as well as the economic impact of the presence of toxins [12,13,14].

Different groups of toxins produce different toxic syndromes that vary substantially in their effects. The saxitoxin group is probably the one entailing the most risk because it causes paralytic shellfish poisoning (PSP), a kind of intoxication that has led to many deaths all over the world [15,16]. The toxins in the okadaic acid (OA) group (OA, dinophysistoxins (DTX) 1 and 2, and their derivatives (7-O-acyl esters and different okadaates)), are much less toxic. They are not lethal, and their acute effects are restricted to gastro-intestinal distresses, causing a syndrome known as diarrhetic shellfish poisoning (DSP) [17,18,19,20]. However, the high persistence of the phytoplankton populations that produce these kinds of toxins in many geographic areas highlights their importance from an economic point of view given that their presence above the legal limit results in a large number of harvesting closures [10,21,22,23].

**Figure 1:**
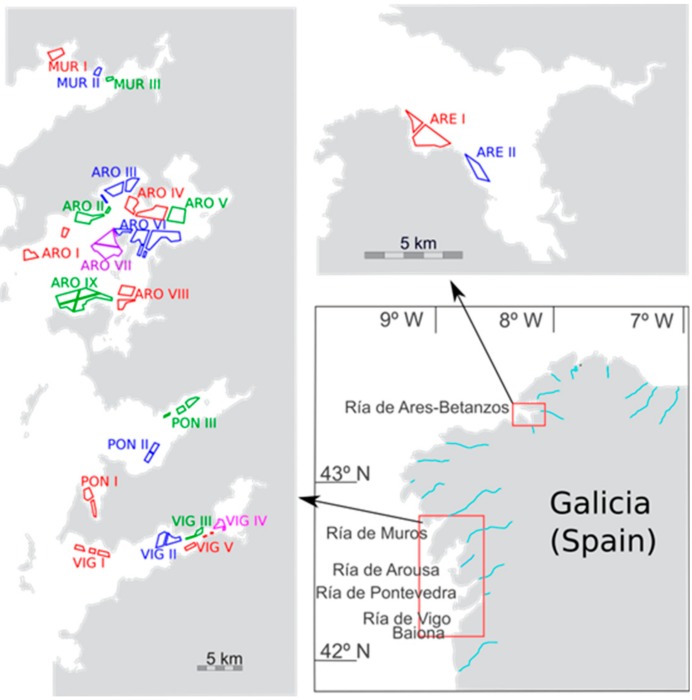
Mussel production areas of Galicia.

In Galicia (Figure 1), an area of intensive mussel (*Mytilus galloprovincialis*) aquaculture (approx. 250,000 *t* per year) as well as the intensive exploitation of natural beds and semi-cultured bivalves (clams, cockles, scallops), an official system has been monitoring PSP toxicity in bivalves since 1977 and DSP, since 1985, after an outbreak of this illness in which over 5000 cases were recorded [10,24]. DSP levels were determined by using the mouse bioassay until the beginning of 2014, and by LC-MS/MS since then. During this period, the days per year that harvesting areas were closed, varied. In some production areas, however, closures often lasted over 200 days [10,21]. The biotoxin monitoring system has been used by Intecmar since 1995.

In the area toxins of the okadaic acid group, (OA and DTX2) have been linked to *Dinophysis* species, as well as to Pectenotoxins [10]. Yessotoxins have been associated with *Lingulodinium polyedra* blooms [25] and *Protoceratium reticulatum* [26].

One of the reasons why the mouse bioassay was replaced by LC-MS/MS as the reference method to identify DSP toxicity in the EU, was its lack of specificity [17]. Not only can the toxins of the OA group yield positive results in the assay, other compounds, such as pectenotoxins, azaspiracids, or yessotoxins—which, due to their polarity, are co-extracted with OA—can also kill the mice with a similar symptomology. It is therefore not possible to distinguish which lipophilic compound produces the positive responses of the mouse bioassay and consequently to be able to apply different legal limits to compounds with different toxicities.

This work aimed to determine the importance of the EU regulated toxins in bivalve market closures in Galicia, their relative importance in the species of commercial interest, their temporal and spatial distribution, the possible biotransformations that they undergo in the bivalves, the uptake and depuration velocities, and finally, to evaluate some aspects of the efficiency of the monitoring system.

## 2. Results

### 2.1. Toxin Profiles

**Figure 2:**
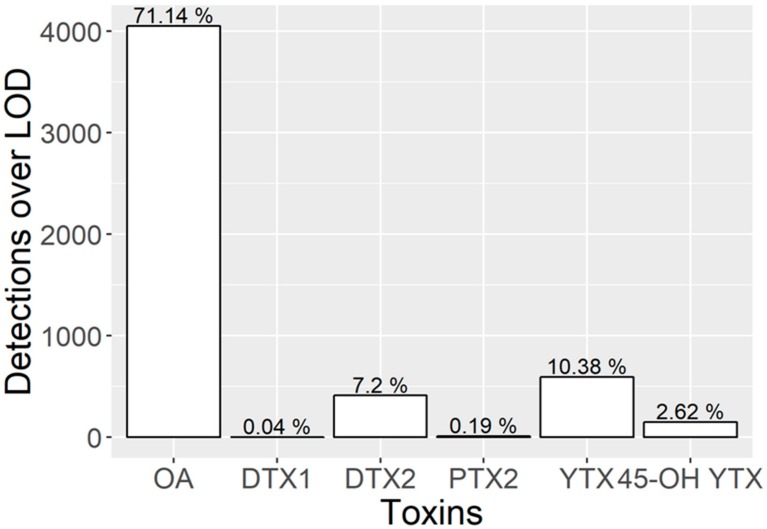
Number of samples in which the toxins were found above the limit of detection (LOD). The numbers above the bars indicate the percentage of the total number of samples analyzed.

Okadaic acid, DTX2, yessotoxin, and 45-OH yessotoxin were the most frequently detected toxins (Figure 2). Pectenotoxin 2 (PTX2) was rarely found (0.18%) and DTX1 was only found in two samples (0.04%). PTX1, HomoYTX, 45OH-homoYTX, AZA1, AZA2, and AZA3 were not found over the limit of detection (1/10 limit of quantification), during the 2014–2017 period.

**Figure 3:**
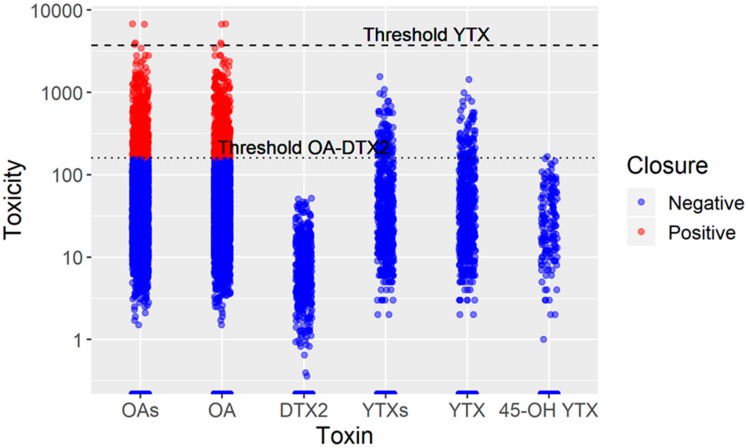
Toxicities (OA-equivalents and YTX-equivalents µg kg–1) of the samples analyzed during the study. OAs is the sum of the toxicities corresponding to OA and DTX2, while YTXs are those corresponding to the sum of YTX and 45-OH YTX. The horizontal lines indicate the legal threshold for harvesting closure.

Toxicity was higher than the EU legal limit in 570 out of the 5561 samples (10.3%) in which toxins of the OA group were quantified after alkaline hydrolysis, with other toxins being quantified without this treatment. In all these cases, toxicity was only due to toxins of the OA group. The toxicity observed was mainly due to OA (10.1%) and only a minor proportion to the combination of OA and DTX2 (0.2%). In no case, did the individual presence of DTX2, YTX, 45-OH YTX, or a combination of the latter two attain levels in the bivalve molluscs that exceeded the EU legal limit (Figure 3).

**Figure 4:**
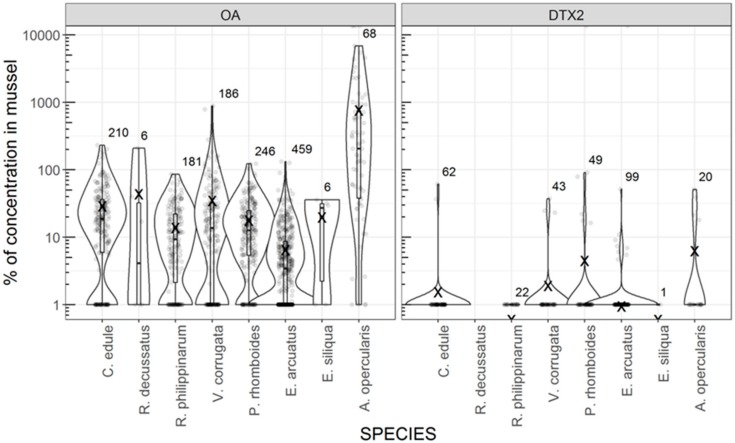
Violin plots of the percentage of OA and DTX2 in several bivalve species in relation to raft mussels from the same area and the same week. The shape of the “violin” shows the distribution of the observations and the box plot inside is standard: quartiles, and median (box), min and max (whiskers). X indicates the mean after removing 6 observations in which the concentration in raft mussel was 0. The numbers of observations and the individual values (dots) are also shown.

**Figure 5:**
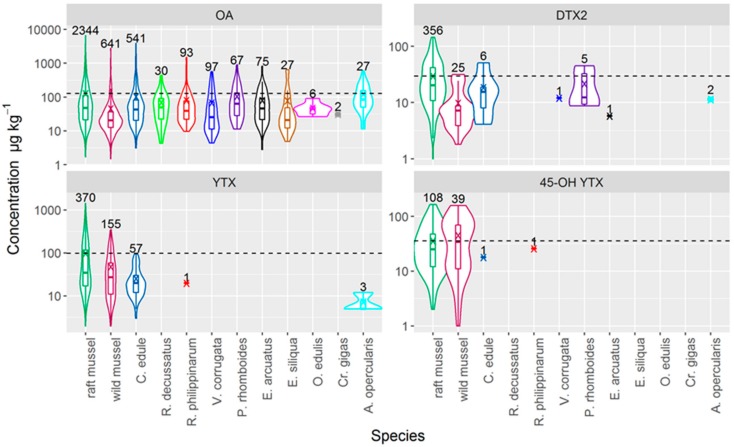
Violin plots of the concentration of OA, DTX2, YTX, and 45-OH YTX in several bivalve species using all available results. The shape of the “violin” shows the distribution of the observations and the box plot inside is standard: quartiles and median (box), min and max (whiskers). The numbers of observations larger than 0 are also shown. The horizontal lines represent the average concentration in raft mussel.

Raft cultured mussels attained higher OA and DTX2 levels than the other species studied, except for OA in *Aequipecten opercularis*. This can be observed when levels in raft mussels are compared with those recorded for other species in the same week and area (Figure 4), and in most cases, when all the observations of each species are included in the comparison (Figure 5). Yessotoxins also seem to be more readily accumulated by mussels than by other species (Figure 5).

**Figure 6:**
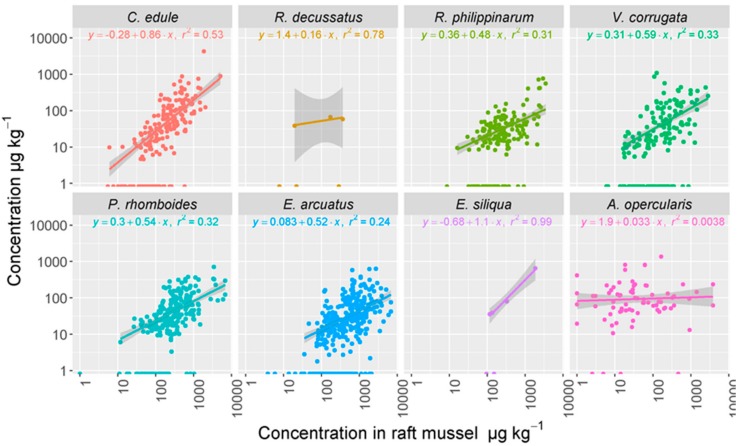
Relationship between OA concentration in raft mussels and other bivalve species. The estimated regression equations are shown. The grey areas represent the 95% confidence intervals for the regression lines.

When OA was detected in mussels, it was sometimes found in other species as well. In these cases, their relationship was linear and positive (Figure 6). OA was always present in mussels when it was detected in other species, with the only exception to this toxin uptake pattern being *A. opercularis* for which no apparent relationship was observed (Figure 6). As expected from the differences in OA concentration between raft mussels and the other species studied, the estimated regression coefficients were lower than 1, except for the razor clam *E. siliqua* (based on only three observations and they were very close to 1). Regarding other lipophilic toxins, the relationship between their concentrations in mussel and the other species studied could not be established because, in most cases, these toxins were only detectable in mussels.

### 2.2. Temporal Variation

**Figure 7:**
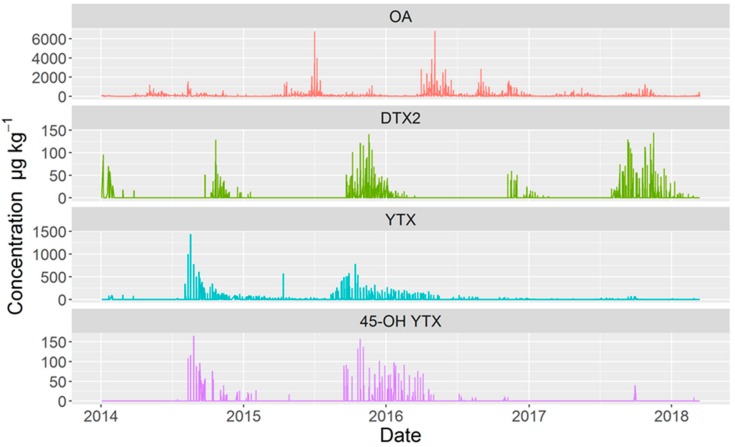
Time course of the concentration of the main toxins during the four years studied.

There was no temporal coincidence in the appearance of YTXs and toxins of the okadaic acid group. Yessotoxin was more frequently detected than 45-OH YTX, but their peaks in abundance took place simultaneously. YTX was always present when 45-OH YTX was detected. Okadaic acid and DTX2 showed a similar, but not identical, pattern. Typically, when DTX2 appeared, OA was always present, but when the most important peaks of OA were recorded, DTX2 was absent (Figure 7).

When the data were analyzed by principal component analysis (PCA), three main components were obtained (Figure S1). The first one is associated with YTX and 45-OH YTX with the toxins of the OA group loadings close to 0 on this component, which indicates that YTXs and OAs appeared independently. In contrast, OA and DTX2 do not covariate as strongly as YTXs and they have intermediate loadings on both the second and third components, indicating that YTXs have a common variation, while OA and DTX2 also behave independently.

### 2.3. Annual and Seasonal Variation

The toxin levels attained in the blooms that took place in the period studied varied with year and toxin (Figure S2). OA levels were highest in 2016, followed by 2015, 2014, and finally in 2017. The proportion of samples having an OA concentration under the LOD (Figure S2) was always low (less than 40%) and showed the same trend as the average levels. The DTX2 pattern was different: 2016 showed the minimum average concentration, while 2017, 2015, and 2014 exhibited similar levels that did not differ statistically from one another (when all samples were considered). The percentage of samples below the LOD for DTX2 was much higher than for OA, which was always above 91%. Yessotoxin concentration showed a decreasing trend during the period studied, with minimum levels recorded in 2017 (all differences between years were statistically significant). As in the case of OA, the percentage of samples below the LOD for YTX was proportional to the average concentration, with the maximum value found in 2014 (79.1%) and the minimum in 2017 (97.7%). 45-OH YTX showed basically the same pattern as YTX, but 2015 and 2016 did not differ significantly.

**Figure 8:**
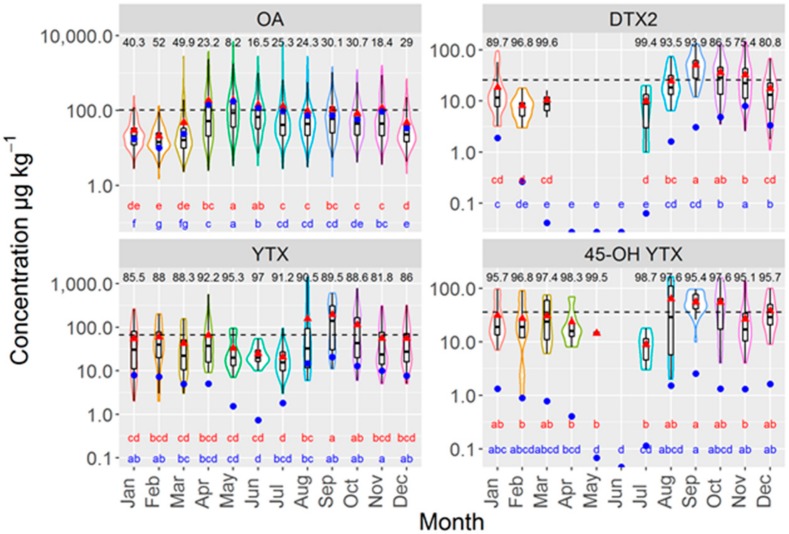
Violin plots of the concentrations of OA, DTX2, YTX, and 45-OH YTX in several bivalve species (“violin description as in Figure 4), including the means corresponding to all observations (blue circles) and observations over the LOD (red triangles). The numbers in the upper part of each plot are the percentages of observations lower than the LOD, and the letters in the lower part are the corresponding Tukey HSD test groups for all observations (red) and observations over the LOD (blue). The horizontal line represents the average toxin concentration.

Maximum OA levels were recorded during the April–July period, and moderate levels were maintained until November, with the subsequent months (from December to March) having minimal OA concentrations (Figure 8). DTX2 showed a different pattern, reaching a maximum in September, later exhibiting a decreasing trend and finally falling to levels below the LOD between April and June. The Yessotoxin maximum took place in September and levels clearly above the mean were also observed for both the previous and following months. The lowest concentrations were recorded in the period between May and July, with the remaining months hovering around the average value (Figure 8). The pattern of 45-OH YTX was the same as the one found in YTX.

### 2.4. Geographical Distribution of the Toxins Detected in Raft Mussels

If the samples with OA levels below the LOD are excluded from the statistical analyses, the intensity of OA episodes was higher than the overall mean for all the production areas in the Rías of Muros and Pontevedra and one area in both the Rías of Arousa and Vigo (Figure S3). Two production areas in the Ría de Pontevedra reached maximum values while the lowest levels were observed in an area from the Ría de Arousa (ARO-VIII). The two production areas in the northernmost Ría (Ría de Ares) also had average concentrations substantially below the overall mean.

When the observations below the LOD were included (measuring the actual impact of the toxins), the same trend was observed, but the lowest concentration corresponded, in this case, to a different area of the Ría de Arousa (ARO-IV).

The intensity of DTX2 episodes showed almost the same geographical pattern as the one observed for OA episodes, with the only difference being a production area from the Ría de Vigo which also attained an average value above the overall mean. When LOQs are included in the computation, the same trend is maintained but, in this toxin, the difference between the two means (whether or not the LOQ is included) are much greater than in OA.

Average values for YTX and 45-OH YTX were only above the general mean in the production areas of the Ría de Ares and, in the case of YTX alone, in the outer area of the Ría de Vigo as well (VIG-I), although in a much lower concentration than the one recorded in the Ría de Ares. When concentrations below the LOQ are included, the general trend was the same but the differences between production areas in the Ría de Ares and all the other Rías were even greater.

**Figure 9:**
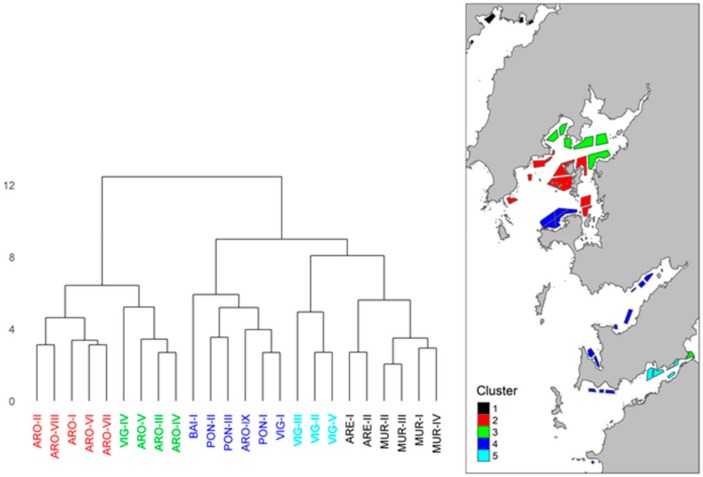
Dendrogram of distances between areas obtained by cluster analysis of the concentrations of OA in the different mussel culture areas of the Galician Rías (A), and distribution of the groups obtained in the Rías Baixas.

When the differences in OA concentration between mussel culture areas every sampled week were studied by cluster analysis (to show how similar the concentrations of OA were during the sampling period), 5 groups could be distinguished (Figure 9). In one of the clusters, all the production areas of the Ría de Pontevedra are grouped together with the outer production areas of the Ría de Vigo (including Baiona Bay) and the Ría de Arousa, but the latter case includes only the southern side. A second cluster groups together the inner areas of the Ría de Vigo and Arousa. A third cluster places all the production areas from both the Ría de Muros and Ares-Betanzos in the same group. Finally, the remaining two clusters group separately the production areas located in the central area of the Ría de Arousa and Vigo.

### 2.5. OA, DTX2, YTXs and their Biotransformations

**Figure 10:**
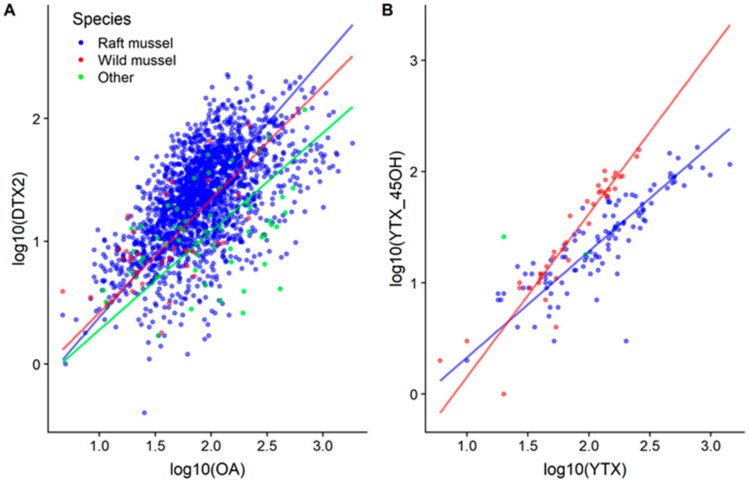
Relationship between OA and DTX2 (**A**) and YTX and 45-OH YTX (**B**), in raft and wild mussels and other species. Lines represent the model II regression lines (standard major axis) corresponding to each group.

When OA and DTX2 appeared together, they showed an almost perfect linear relationship (a slight asymptotic shape can be observed), with no clear difference between species or (mussel) habitat (Figure 10). The average levels of the ratio OA/DTX2 were 5.7, 5.4, and 14.4, in raft mussels, wild mussels, and other species, respectively.

YTX and 45-OH YTX were also linearly related, but for this pair of toxins, the relationship was clearly affected by the mussel habitat, with the proportion of 45-OH YTX being higher in wild mussels than in raft cultured ones (Figure 10). For other bivalve species, there are not enough data to reliably compare them.

**Figure 11:**
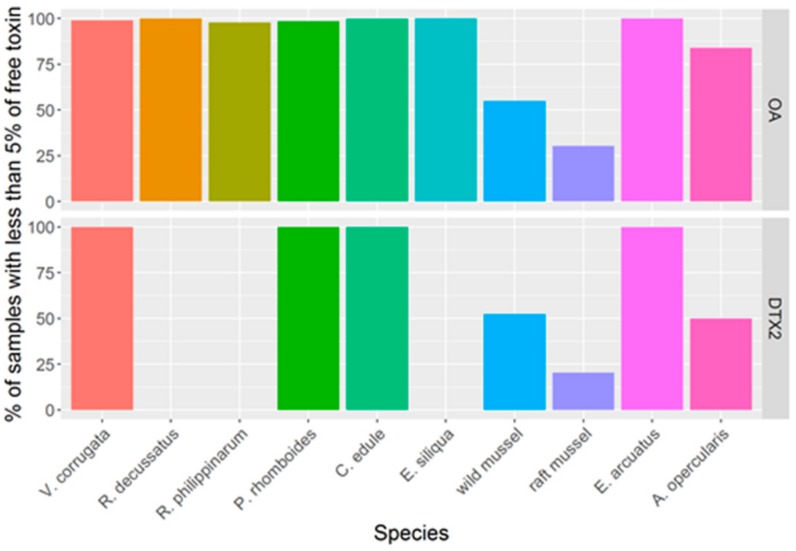
Percentage of the samples in which the proportion of free toxins was less than 5% of the total.

OA and DTX2 were found to exist as free and esterified forms in the bivalves studied. The degree of esterification varied with both the species and the toxin. For clams and cockles, in nearly all samples analyzed, the esterified toxins constitute more than 95% of the total toxin content. In mussels (raft cultured and wild) and *A. opercularis*, the number of samples with such a high proportion of esterified toxins was lower (especially in raft mussels) than what was observed for the other bivalve species. The degree of esterification observed was different for the two toxins; it was higher in OA than in DTX2 (Figure 11).

### 2.6. Timing of Episode Onset

**Figure 12:**
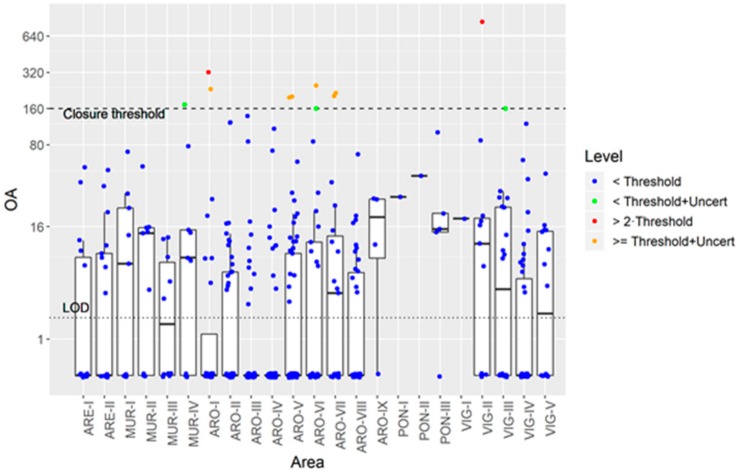
OA concentration (µg kg^−1^) attained in areas where in the previous week its concentration was below the limit of detection (LOD).

The changes in OA concentration between samplings between consecutive weeks were examined. When the OA levels in the first week were below the detection level (LOD), most of the samples corresponding to the second week, 97.7% (469 samples), were below the closure threshold (Figure 12). A small percentage, 0.4% (2 samples), were twice as high as the threshold and 1.9% were between these two levels (9 samples), 3 of which were within the uncertainty range of the analytical method for the closure level. The presence of samples reaching levels above the regulatory level was more frequent in the Ría de Arousa (8 out of the 11 samples) than in the other Rías.

### 2.7. Apparent Uptake and Depuration Rates by Cultured Mussel

#### 2.7.1. Differences between Toxins

The two toxins for which the apparent uptake and depuration rates () were studied (OA and DTX2) behave similarly. In both cases, the apparent rates were due to the fact that even when one of the processes dominates (uptake or depuration), the two take place simultaneously. Both the average uptake and depuration rates were quite similar (around 0.1 duplications · day^−1^), and only the uptake of DTX2 seems to have taken place at a slightly lower rate as compared with OA (Figure S4).

#### 2.7.2. Differences as Compared with Culture Areas

##### Okadaic Acid

**Figure 13:**
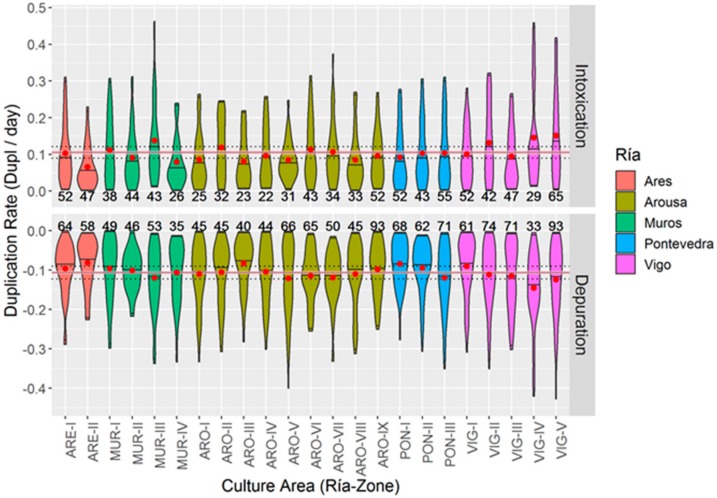
Violin plots of the duplication rates of OA in the mussel culture areas after removing outliers. Means = red circles, the pink ribbon indicates the overall mean ± 2·SE, and the blue ribbon indicates the overall mean ± 15%. Numbers above and below the “violins” are the actual number of observations.

The velocities of uptake and elimination of okadaic acid were, on average, quite constant in the different areas studied. Most of the means fall within the ± 15% interval of the overall mean (Figure 13). In general, uptake and depuration rates were slightly, but directly, related (*p* = 0.0016, r^2^ = 0.38). Both depuration and uptake are around 0.1 duplications/day, meaning that typically the values observed one week are increased or decreased by a factor of approximately 1.6 (2^0.1*7^) the next one. The maximum estimated duplication rates, excluding outliers, are around 0.45, and the values in most areas did not exceed, 0.3 duplications/day, which indicates change factors of 8.9 x and 4.2 x, respectively.

The highest average (and also maximum) uptake rates were recorded in the inner areas of the Ría de Vigo (VIG-IV and VIG-V) and the Ría de Muros (MUR-III). These areas were also the ones with the highest depuration rates. The lowest average uptake rates were recorded in the inner production area of the Ría de Ares (ARE-II) and the outer area of the Ría de Muros (MUR-IV). However, in this case, only ARE-II was among the areas identified as having the lowest depuration (along with PON-I and ARO-III, from the inner and middle areas of the Rías de Pontevedra and Arousa, respectively).

##### DTX2

The uptake rates observed in DTX2 were similar to the ones found for OA in absolute value, but substantially different in terms of spatial variation. The highest average uptake rates were found in the inner area of the Ría de Pontevedra, followed by the middle area of the same Ría and by the southwestern zone of the Ría de Arousa (Figure 13). The direct relationship between uptake and depuration rates, which was found for OA, continues to apply to this toxin (*p* = 0.003, r^2^ = 0.43).

## 3. Discussion

### 3.1. Incidence of Marine Toxins in Galician Production Areas

Okadaic acid is the toxin with the highest incidence in Galician production areas, followed by its isomer DTX2 and by two compounds of the yessotoxin group, YTX and 45-OH YTX. All the other toxins monitored (among those regulated by the EU) rarely or seldom appeared. Yessotoxins have always been well below their legal limit, and thus did not contribute to any harvesting closures. OA was clearly the most prominent toxin, found to be responsible for 98% of the closures due to lipophilic toxins. DTX2 alone was never responsible for closures but it did contribute to the remaining 2% since its toxicity must be added to that of OA. PTX2 and DTX1 have been recorded only in isolated samples and PTX1 has never been found. Azaspiracids have only been identified at trace levels, as described in a previous work [27]. Other non-regulated toxins, such as 13-desmethyl spirolide C, have been found in the water column and sediment [28,29] but, like the AZAs, only at trace levels. Pinnatoxin G has been detected in different bivalve molluscs with concentrations generally below 3 µg·Kg^−1^ although with a maximum of 15 µg·Kg^−1^ [30].

The profiles described in this study are quite similar to those found in Portugal [31] where two toxins of the OA group, OA and DTX2, were identified as the main toxins responsible for harvesting closures. PTXs and AZAs were always, or almost always, at trace levels, and YTXs appeared at appreciable levels, but below the regulatory level. In Andalusia (SW Spain) [32], OA was also the main toxin, sometimes accompanied by DTX2. PTX2 was detected at low or trace levels, as well as AZAs, except during a bloom of *Amphidoma languida* which took place in 2009 [33], and yessotoxins were found mostly at trace levels [32]. In Great Britain, the toxins of the OA group were also the most prevalent (from 2011 to 2016) [22] with PTX2 quantified only on rare occasions. The most relevant difference between this area and the Atlantic coast of the Iberian Peninsula is the presence (relatively frequent at low concentrations) of DTX1 [34]. In Ireland, DTX2 was the main toxin present during DSP episodes associated with *D. acuta* [35,36].

The ratio between total OA and total DTX2 was lower in mussels (approx. 5.5) than in the other bivalve species studied (14.4). Taking into account that these toxins cannot interconvert, the differences observed probably stem from differences in their uptake or depuration or from the relationship between the ability of toxins to carry out esterification and depuration [3]. The sampling bias, introduced by the fact that other bivalves are only sampled after mussels have been shown to exceed the legal toxicity threshold (see material and methods section), cannot be responsible for the differences observed. The reason is that DTX2 is depurated from mussels at a lower rate than OA, and consequently the OA/DTX2 ratio should be higher during the initial period of each *Dinophysis* bloom (when mussels are sampled, but not other species). The ratio in mussels is higher than those found in other studies for the same area [37], for Portugal [38,39,40,41], or for the main species responsible for its production in this and other areas of Europe, *Dinophysis acuta* [10,35,41,42,43]. It is important to highlight the fact that the OA/DTX2 ratio obtained in this study is the mean result of at least eight DSP episodes (spring and autumn episodes for each year) and it will depend, on each moment of the *D. acuminata*/*D. acuta* ratio studied. In this sense, in northwest Spain, the spring DSP episodes are mainly due to *D. acuminata,* which yields only OA [10], while some years autumn episodes are caused only by *D. acuminata* and other years, by a combination of both *Dinophysis* species in varying proportions [44,45,46,47]. In strains of *D. acuta* from Galicia, a 3:2 ratio of OA:DTX2 has been measured [10]. However, in Ireland, for instance, the same species may have a 3:4 ratio, as they can be calculated from isolated cells or phytoplankton concentrates [48,49]. 

Pectenotoxin 2 (PTX2) was clearly underrepresented in bivalves in relation to the typical content of the producer species, *D. acuta,* from Galicia, Portugal [10,50,51,52] and other areas such as Great Britain [22], Norway [53,54], and Ireland [36]. This is also true of the proportions between OA and PTX2 in passive samplers [45,55]. The main reason for this underrepresentation may very likely be attributed to the fast conversion of PTX2 to its seco-acid and of this compound to several esters (with fatty acids of different carbon chain lengths) [3,38,39,55,56,57,58,59,60].

The main toxins are transformed to a different extent by the bivalve species studied. OA and DTX2 are esterified to 7-O-acyl esters (“DTX3”) almost completely by most bivalve species. In mussels and *A. opercularis*, however, this esterification is not complete. Moreover, in these species, DTX2 was esterified in a lower proportion than OA.

As already discussed, PTX2 is transformed to PTX2-seco acid which is esterified with fatty acids. Although the products of these transformations were not analyzed in this study, they are quite likely to be responsible for the differences in accumulation observed between OA/DTX2 and PTX2.

Yessotoxin in mussels was partially transformed into 45-OH YTX, which was consistent with previous results [61,62]. In other species, 45-OH YTX was hardly detected at all, in part because YTX was not accumulated in most of the species studied, but in the one where it was accumulated (the cockle *Cerastoderma edule*), this was probably because its transformation to 45-OH YTX either did not take place or did so at a substantially lower rate than in mussels. In wild mussels, the oxidation of YTX to 45-OH YTX seems to have been faster than in raft cultured ones. Taking into account that in both cases the mussel species is the same, differences in metabolic rates would be responsible for this discrepancy, which is striking because raft mussels are always immersed (and consequently always getting oxygen from the water), whereas wild mussels are out of the water during part of the day (two semidiurnal tides in the area), with no oxygen available during these periods.

### 3.2. Incidence of Toxins in Commercially Important Bivalve Species

In general, raft cultured mussels were the most seriously affected bivalves, with perhaps the exception of *A. opercularis*. For okadaic acid and DTX2, a direct comparison of the concentrations attained during the same week in raft mussels with those of other species inhabiting the same area, showed that they reached the highest average levels of DTX2, and with the exception of *A. opercularis*, of OA as well. For *A. opercularis,* this comparison might be unreliable since this species has high swimming capabilities and consequently, the organisms captured in one area might easily have been exposed to the toxins in other areas. When the concentrations recorded in all the samples studied are considered, raft mussels had the highest average concentrations of all regulated toxins, and particularly yessotoxins, as reported in France (Normandie) where YTX was detected in mussels but not in oysters or clams [63]. *Aequipecten opercularis* seems to accumulate OA approximately at the same concentrations as raft mussels, but not any other toxin. Mussel is also the species that accumulates more toxins of the okadaic acid group in Portugal (which shares the most commercially important bivalve species with Galicia) [31,39,40].

The concentrations of OA in most species were linearly related to those in raft mussels, with regression slopes that were generally below 1. There were also some observations in which the toxins were detected in mussels but not in the other species. This fact, together with the information on average levels discussed above, confirms that raft mussels are a good indicator species for this area since it has been used since this monitoring system was designed (Mariño et al., 1998). In terms of average levels, *Aequipecten opercularis* behaves differently, having OA concentrations that are practically independent of those found in raft mussels, which therefore cannot be used as an indicator for this species. The most likely cause of this non-existent relationship might be, as discussed earlier, the swimming capability of the individuals of this species. In Scotland, the levels of OA found in *A. opercularis* were much lower than those found in mussels [23]. Different mussel species are traditionally used as sentinel organisms, especially for PSP toxins, because they are known to accumulate these substances faster than other species (Shumway et al. 1995; Anderson et al., 2001). But mussels are also known to be the best indicator of the accumulation of OA group toxins [64,65,66,67].

### 3.3. Temporal Variation and Patterns

Yessotoxins and toxins of the okadaic acid group followed different temporal dynamics. The two yessotoxins studied covariated mostly, with YTX, driving the dynamics and following this was 45-OH, but at a lower concentration. The two toxins of the okadaic acid group had a markedly different temporal evolution. When DTX2 appeared, OA was always present but the inverse was not true. This is clearly due to the toxin profile of the two main *Dinophysis* species in the area—*D. acuta* and *D. acuminate*—because the former contains OA and DTX2, while the latter contains only OA [10,68,69,70].

There was no appreciable trend in OA and DTX2, during the four years studied, but a decreasing tendency would seem to exist in YTXs. In any case, the time series is too short to make any generalizations in this respect.

The seasonal variation was clear, with maxima of OA in spring, DTX2 in autumn, and YTXs in summer-early autumn. The seasonal variation of the OA group of toxins is what would be expected from the typical annual cycle of *Dinophysis* on the Atlantic coast of Europe, which is characterized by blooms of species of the *D. acuminata* (producer of OA) complex during spring and summer followed by *D. acuta* (producer of OA and DTX2) [10]. The same pattern has been recorded in the other areas that make up the Atlantic border of the Iberian Peninsula (Portugal and Andalusia) [32,38]. A similar variation was found in Ireland [71], but with the OA maximum taking place in summer instead of spring. The detection of YTX episodes in summer in the area studied has been reported and associated with *Lingulodinium polyedra* [25] and *Protoceratium reticulatum* [26].

The economic impact of these blooms is variable, resulting in maximum values when they take place in (or last until) the months in which the demand for bivalves is highest, namely, July and December [72].

### 3.4. Spatial Variation 

Two Rías (Pontevedra and Muros) are more affected by OA and DTX2 than the mean. As discussed in the material and methods section, the fact that the sampling frequency in an area was reduced when the toxicity was well over the legal threshold, would cause the differences observed to underestimate the real ones. The inner areas of the Rías generally exhibited a lower incidence of these toxins than the outer zones, suggesting that, in most cases, toxic populations are advected to the Rías. It is known that the outer and middle areas of the Rías Baixas are clearly dominated by oceanic characteristics, especially in spring and summer, and only the inner zones actually behave like an estuary [73,74]. In terms of similarity in the timing and intensity of the episodes (using OA as variable), the areas studied can be grouped by cluster analysis into 5 different zones. One of these groups most of the outer areas of the Rías and the whole Ría de Pontevedra together, which would suggest that the outer zones are affected by the shelf waters, while the inner areas are able to maintain local phytoplankton populations. Another cluster that groups the innermost zones of the Rías de Arousa and Vigo together, represents the areas with the lowest impact of toxins. The other groups represent particular areas of each Ría that are probably conditioned by the hydrographic conditions during downwelling events, which generate frontal areas in the central portions of the Rías [75,76,77].

### 3.5. Intoxication, Depuration of OA, and the Monitoring System Risk

When a toxic episode starts, there is always the possibility that the organisms being monitored accumulate toxins above the legal limit in the period between two consecutive samplings, which is why a weekly sampling for toxin analyses in live bivalve molluscs was established in the EU legislation [78]. In the Galician monitoring system, the probability of this happening is low, but not negligible (about 2%). The risk for the consumers is not high given that a 3x safety factor is included in the regulatory limit [79] and none of the samples exceeded this threshold. This would suggest that the procedures (action plans) established to make decisions on sampling frequency at each phase of each toxic episode are suitable for the intended purpose. As the risk is clearly associated with certain areas (most of the cases detected have been found in the Ría de Arousa), the obvious cause would be the phytoplankton and oceanographic conditions. Therefore, a detailed study of these two aspects should always be considered in a monitoring system. The Galician monitoring system uses this kind of information to minimize risk, and precautionary measures are routinely taken when toxic phytoplankton populations and oceanographic conditions tend to favor the intoxication of bivalves. The most important of these are cautionary closures which are put into effect until a new mussel sample is analyzed and sampling is intensified, as set out in the Commission Implementing Regulation (EU) 2019/627 [80].

Upon detection of the first sample testing positive for okadaic acid, the velocity at which the intoxication proceeded differed depending on the area. Our estimates indicate that over the course of two consecutive weeks the levels changed, on average, by a factor of 1.6 x, with maxima of 8.9 x and 4.2 x for most locations. In Galicia’s monitoring system, sampling frequency is increased (to several samples per week) when toxins are detected in the bivalves, following an established action plan [81], which includes cautionary closures mostly when advection of toxic populations to the culture areas is suspected (as used, for example, during a bloom of *D. acuminata* [82]).

Surprisingly, and in concordance with what has been observed in Andalusia [32], the estimated apparent depuration rates are similar to the intoxication rates. Moreover, both OA and DTX2 have similar apparent rates, although it has been experimentally demonstrated that the true depuration rate of DTX2 is much lower than that of OA [37,38,39,53] (and this has been observed during the final steps of several episodes). The most likely explanation is that the apparent depuration is determined more by the rate at which the toxic plankton populations disappear from the water column than by the true depuration of the toxins from the bivalve tissues. This would explain the relationship between the apparent intoxication and depuration rates because, in both cases, the dynamics of the water (advection, diffusion, etc.) would regulate the velocity at which the concentration of toxic algae changes in the area.

## 4. Conclusions

Okadaic acid was the main lipophilic toxin in the area. It was the only one that produced harvesting closures. DTX2, YTX, and 45-OH YTX were also found at quantifiable levels, but well below their corresponding regulatory limits. Raft mussels were the organisms most affected by lipophilic toxins, followed by wild mussels, cockles, and the scallop *Aequipecten opercularis*. OA levels in raft mussels and other species were well correlated (except *A. opercularis*), which would support its use as an indicator. Spatially, the most northern Ría (Ría de Ares) behaved differently from the southern ones (Muros to Vigo), with the importance of OA group toxins being relatively low, but higher for YTXs in the former. The outer areas of the Rías generally share their toxin concentration, as do the inner parts of some Rías. The middle zones, however, behave in different ways. The OA level reached a maximum in spring and a second (less pronounced) maximum in autumn. DTX2 was practically restricted to the fall–winter season. YTXs peaked in August–September. The inter-annual variability was important, especially for YTXs.

The toxins of the OA group were biotransformed. They were esterified with fatty acids to 7-O-acyl esters, to almost 100% in most species, but not in mussels and *A. opercularis*. In these species, DTX2 was less esterified than OA. YTX was transformed into 45-OH YTX in mussels, but this process was not detected in other species.

Apparent uptake and depuration velocities were found to be similar as well as related. In general, locations with a high intoxication velocity also undergo rapid depuration. This suggests that the two rates are regulated by the availability of toxic plankton in the area.

The monitoring system was quite efficient in the management of the harvesting closures. In only 8 cases out of 480 in which OA was detected after being below the detection limit in the previous sampling, did its concentration exceed unequivocally the legal limit. Only two cases were found to be twice as high as the limit concentration. However, they did not pose a significant risk to human health, taking into account that a safety factor of 3x was used to establish the allowable limit and that self-checking measures must be implemented by the distributors and food processing companies.

## 5. Material and Methods

### 5.1. Sampling

Samples for analysis by LC-MS/MS were collected from January 2014 until December 2017, with a frequency determined by the action plan of Intecmar—a minimum of once per week in each production area when harvesting is allowed. Mussel samples were taken from three depths on the raft culture ropes from 26 production areas. In most cases, the samples of the three depths were combined into one. Moreover, wild mussels were collected weekly from 16 areas where there are wild populations of different bivalve species but no mussel culture on rafts.

Other bivalve species were only sampled when any type of EU regulated toxin was detected in mussels. As a result of this sampling strategy and of the local abundance of the different species studied, mussel was the most represented species in the samples, followed by the cockle *Cerastoderma edule* and the carpet shell clam *Venerupis corrugata*. Other species were analyzed only on a few occasions (razor clams *Ensis siliqua* and *Ensis arcuatus*, clams *Polititapes rhomboides* and *Ruditapes philippinarum*, and oysters *Magellana gigas* and *Ostrea edulis*.

A total of 18,862 samples were obtained. All of these were analyzed to detect total OA, DTX1, and DTX2 but only a subset of 5561 samples were analyzed for all EU regulated toxins, corresponding to different species (Table S1).

### 5.2. Analysis

#### 5.2.1. Extraction and Hydrolysis

Toxins were extracted from the bivalves following the standardized operating methods of the EU Reference Laboratory consisting of opening the bivalves and discarding their shells. Between 100 g and 150 g of meat was homogenized and a 2 g subsample was taken.

The subsample was extracted with 9 mL of MeOH by vortexing the suspension for 1 min. After centrifuging the mixture at 2000× *g* for 10 min, the supernatant was withdrawn and the pellet resuspended in 9 mL of MeOH. After centrifugation under the same conditions, the new supernatant was withdrawn, combined with the previous one, and the resulting extract was made up to 20 mL with MeOH.

In all cases (18,862 samples), the total concentration of toxins of the OA group was determined by means of introducing a step from alkaline hydrolysis previous to the analysis of the extract following this procedure: 625 µL of 2.5M NaOH was added to a 5 mL aliquot of the methanolic extract; the mix was vortexed for 30 s, and heated at 76 ± 4 °C for 40 min; the hydrolysate was allowed to reach room temperature, weighed in order to check that there were no solvent losses by evaporation, neutralized by adding 625 µL of 2.5M HCl, and vortexed again. An aliquot of the neutralized hydrolysate was filtered through a 0.22 µm syringe filter, and diluted 5/8 (with MeOH) [83]. In a subset of the samples (5561), the extract was not subjected to hydrolysis and the free form of the toxins of the OA group, plus the other EU regulated toxins, were analyzed.

#### 5.2.2. LC-MS/MS

The method used in this study was optimized and validated at Intecmar following the guidelines provided by the SOP of the EU Reference Laboratory for the determination of marine lipophilic toxins in molluscs by LC-MS/MS version 4 [83], and based on the methods described by Gerssen et al. [84,85], and Regueiro et al. [86]. The method is accredited following standard UNE-EN ISO/IEC 17,025 (Accreditation N° 160/LE 394).

Chromatographic separation was carried out using an Acquity UPLC BEH C18 column (2.1 × 100 mm, 1.7 µm) (Waters, Barcelona, Spain), at 45 °C, with a binary gradient of the mobile phase. Mobile phases A and B were 6.7 mM NH4OH (pH11) and MeCN 90% with 6.7 mM NH4OH. The gradient started with 25% B that was maintained for 0.66 min, followed by a linear increment to reach 95% B at minute 3.3, and by maintaining this composition until minute 5.28. The composition was then returned linearly to the initial one in two minutes and maintained until minute 9. Three LC systems were used: one Waters Acquity UPCL system (coupled to a Waters Xevo TQ MS) and two Waters Acquity H-class (coupled to a Waters Xevo TQ-S).

The toxins were detected by means of three triple quadrupole mass spectrometers: one Xevo TQ MS (Waters, Barcelona, Spain), and two Xevo TQ-S, all equipped with ESI interfaces. The systems were operated in positive and negative ionization modes. In the first case, the capillary voltage used was 1V and in the second, 2V. All other operating parameters of the source were the same for both MS models: 450 °C solvation temperature; 850 L·h^−1^ solvation gas flow, and 150 L·h^−1^ cone gas flow. The transitions corresponding to the toxins studied, as well as their limits of detection (LOD) and quantification (LOQ) in bivalve tissues, are given in Table 1.

### 5.3. Computation and Dataset Limitations

The dataset is biased for several reasons. The first stems from the fact that raft-cultured mussels are used as indicator species. So, in many cases, other bivalves are only analyzed after detecting toxins in mussels, and, consequently, the number of observations below the LOD is reduced and the impact of the toxins in those species, overestimated. A second reason is that several LS-MS/MS systems have been used. Although the LOD established for each toxin was common to the three systems, their sensitivities are different and therefore there is a probability that the toxins may not be detected in some samples with true levels over the LOD. These differences should not introduce any bias, as the sample assignation to each system is basically random. A third reason is that, due to operational and economic considerations, when bivalves in an area attain very high toxin concentrations, sampling of that area is stopped for a period of time (taking into account that OA and DTX2 are not eliminated by mussels at rates higher than 0.2 day^−1^ [3]). This procedure means that the absolute concentration maxima are usually not recorded, and consequently, the maximum concentrations in this study underestimate the actual ones.

Some means have been computed using all data (including determinations below the LOD, which are assumed to be 0). In the case of raft mussel, which is sampled every week, this mean gives us an idea of the average impact of the toxin on the species. However, in other species, which are not usually sampled until the toxins are found in raft mussels, this mean value is in fact an overestimation of the actual impact.

When the means were computed excluding the observation below the LOD, they represent the average intensity of the toxic episodes. In this case, they have the same meaning in raft mussel and other species.

Production areas do not have exactly the same geographical boundaries for raft mussels as for scallops or other (typically infaunal) bivalve species, but it is possible to correlate the different sampling points for monitoring raft mussels with each production area of infaunal bivalve molluscs. Raft mussel areas are much more subdivided than the equivalent areas for infaunal species and more than for scallops. In order to compare mussels with the other species, the values obtained in all the individual area subunits of raft mussels corresponding to each area of the bivalves compared, were averaged.

All the comparisons were made on a weekly basis. In the week in which several samples were taken, the maximum level was selected.

When performing a cluster or principal component analysis that uses data from individual areas in the different weeks sampled, some missing combinations appeared due to the lack of available samples for different reasons. Eliminating all records with incomplete observations would make the analysis impossible, so data imputation of the missing observations was used (see packages in the Statistical Analysis section). Notwithstanding, the areas with more than 4 missing data were discarded.

Toxin duplication rates were computed using the toxin concentrations measured in raft mussels, by the expression *(log_2_ (Tox_t0_) − log_2_(Tox_t1_))/(t1 − t0)*, where *Tox* is the concentration of the toxin, and *t1 − t0* the difference (in days) between the time of a sampling and that of the previous one. These duplication rates are, in fact, not real but apparent, because it is the balance of uptake and depuration that is estimated and not each process independently. When uptake dominates, the apparent uptake rate is obtained and when the opposite occurs, the apparent depuration rate is had.

Although under the Galician monitoring system all observations over the LOD are recorded, and used for internal operations, in the official reports only data at or above the LOQ are included.

### 5.4. Statistical Analysis

All statistical analyses have been carried out with R [87], and the following R packages: ggplot2 [88] for general plotting, FactoMineR and FactoExtra for principal component analysis (PCA), mice [89] missMDA [90] for missing data imputation for cluster analysis and PCA, respectively, hclust (core R) and dendextend [91] for cluster analysis, agricolae [92] for ANOVA and TukeyHSD tests, and multcomp [93] for Dunnet tests, and smatr [94] for standard major axis (model II) regression.

## Figures and Tables

**Table 1:** Transitions and quantification limits for the studied toxins.

Toxin	ESI	Precursor Ion (*m/z*)	Product Ion (*m/z*)	Collision Energy (eV)	Cone Voltage (V)	LOQ	LOD
OA	-	803.5	255.2 *	46	86	40 µg OA-eq·kg^−1^	2 µg OA-eq·kg^−1^
			113	56	86		
DTX2	-	803.5	255.2 *	46	86	24 µg OA-eq·kg^−1^	2 µg OA-eq·kg^−1^
			113	56	86		
DTX1	-	817.5	563.4	43	86	40 µg OA-eq·kg^−1^	2 µg OA-eq·kg^−1^
			255.2 *	48	86		
YTX	-	570.4	467.4 *	30	45	0.06 mg YTX-eq·kg^−1^	0.001 mg YTX-eq·kg^−1^
			396.3	35	45		
45OH-YTX	-	578.4	467.4 *	30	45	0.06 mg YTX-eq·kg^−1^	0.001 mg YTX-eq·kg^−1^
			396.4	30	45		
HomoYTX	-	577.5	474.4 *	30	48	0.06 mg YTX-eq·kg^−1^	0.001 mg YTX-eq·kg^−1^
			403.4	30	48		
45OH-homoYTX	-	585.5	403.4	30	45	0.06 mg YTX-eq·kg^·1^	0.001 mg YTX-eq·kg^−1^
			474.4 *	30	45		
PTX1	+	892.5	839.5	25	36	40 µg OA-eq·kg^−1^	0.4 µg OA-eq·kg^−1^
			821.5 *	25	36		
PTX2	+	876.5	841.5	20	36	40 µg OA-eq·kg^−1^	0.4 µg OA-eq·kg^−1^
			823.5 *	25	36		
AZA1	+	842.5	654.5 *	55	50	40 µg AZA1-eq·kg^−1^	4 µg AZA1-eq·kg^−1^
			362.2	50	50		
AZA2	+	856.5	672.5 *	45	27	42 µg AZA1-eq·kg^−1^	4 µg AZA1-eq·kg^−1^
			654.5	45	27		
AZA3	+	828.5	640.4	55	47	41 µg AZA1-eq·kg^−1^	4 µg AZA1-eq·kg^−1^
			362.2 *	55	47		

## Supplementary Materials

The following are available online at https://www.mdpi.com/2072-6651/11/10/612/s1, **Figure S1**. Principal component analysis of the observations using the toxins as variables; **Figure S2**. Violin plots of the concentration of OA, DTX2, YTX and 45-OH YTX in the four years studied; **Figure S3**. Violin plots of toxin concentrations in different mussel culture areas; **Figure S4**. Violin plots of the estimated duplication rates of the toxin concentration in all the studied area; **Figure S5.** Plots of the duplication rates of DTX2 in the mussel culture areas after removing outliers; **Table S1**. Number of samples in which all the EU regulated toxins were analysed, and numbers of samples with all toxins below and over LOD, and below and over the regulatory limit.
