# Supplementary material for: Lipophilic Toxins in Galicia (NW Spain) between 2014 and 2017: Incidence on the Main Molluscan Species and Analysis of the Monitoring Efficiency
Source: Toxins (Basel). 2019 Oct 22;11(10):612. doi: 10.3390/toxins11100612 (PMC6832414; doi:10.3390/toxins11100612)
Supplement: Supplementary file 1 [file toxins-11-00612-s001.pdf]

Supplementary Materials: Lipophilic Toxins in Galicia (NW Spain) between 2014 and 2017: Incidence on the Main Molluscan Species and Analysis of the Monitoring Efficiency

Juan Blanco, Fabiola Arévalo, Jorge Correa and Ángeles Moroño

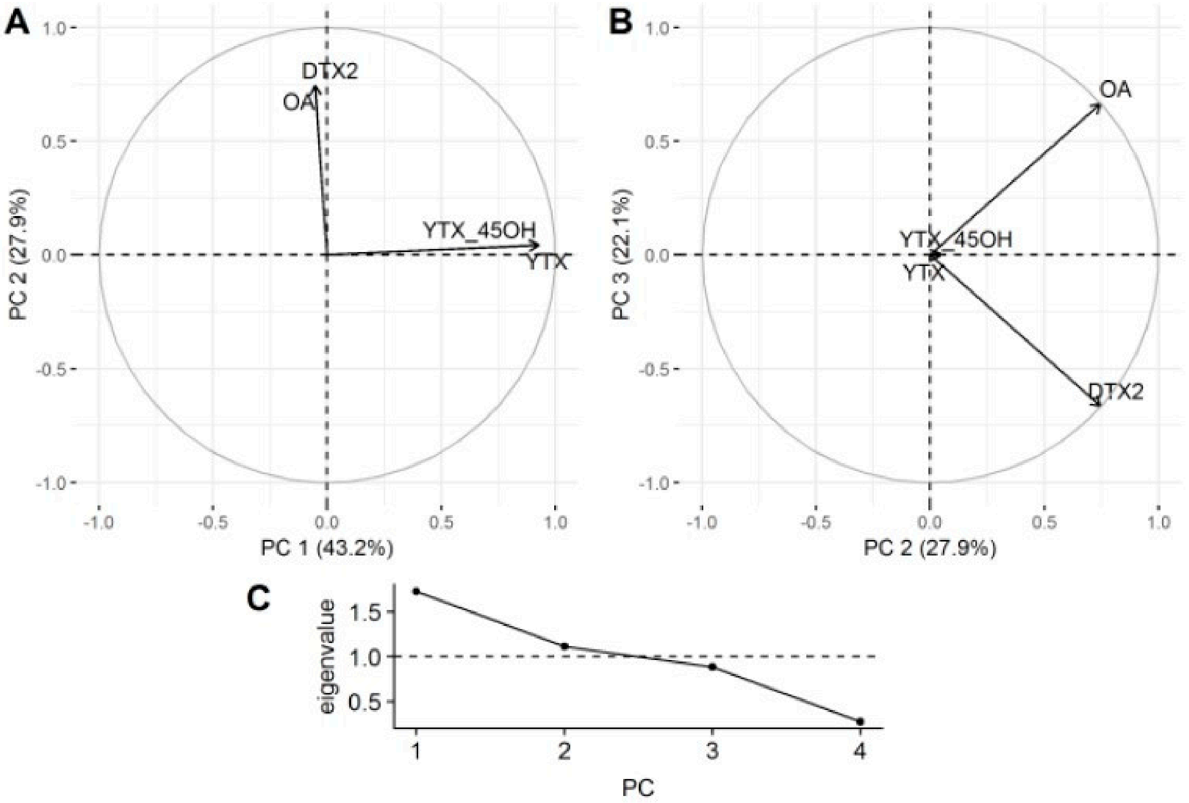

Figure S1. Principal component analysis of the observations using the toxins as variables.

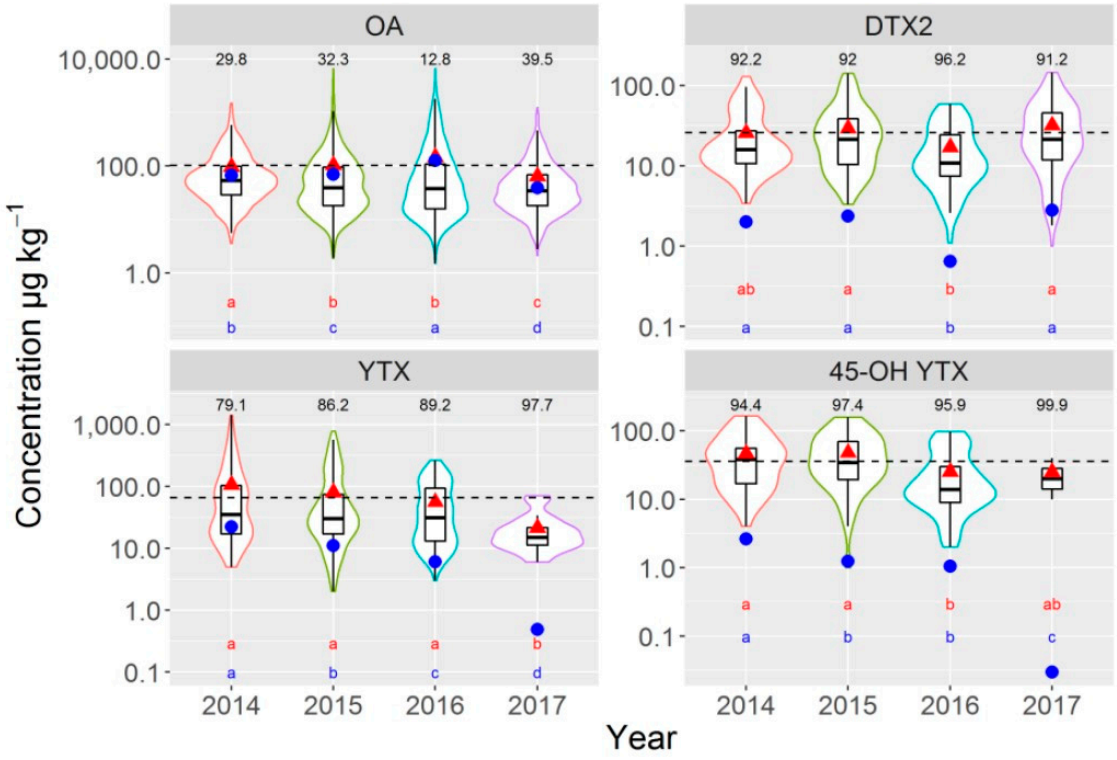

Figure S2. Violin plots of the concentration of OA, DTX2, YTX and 45-OH YTX in the four years studied.

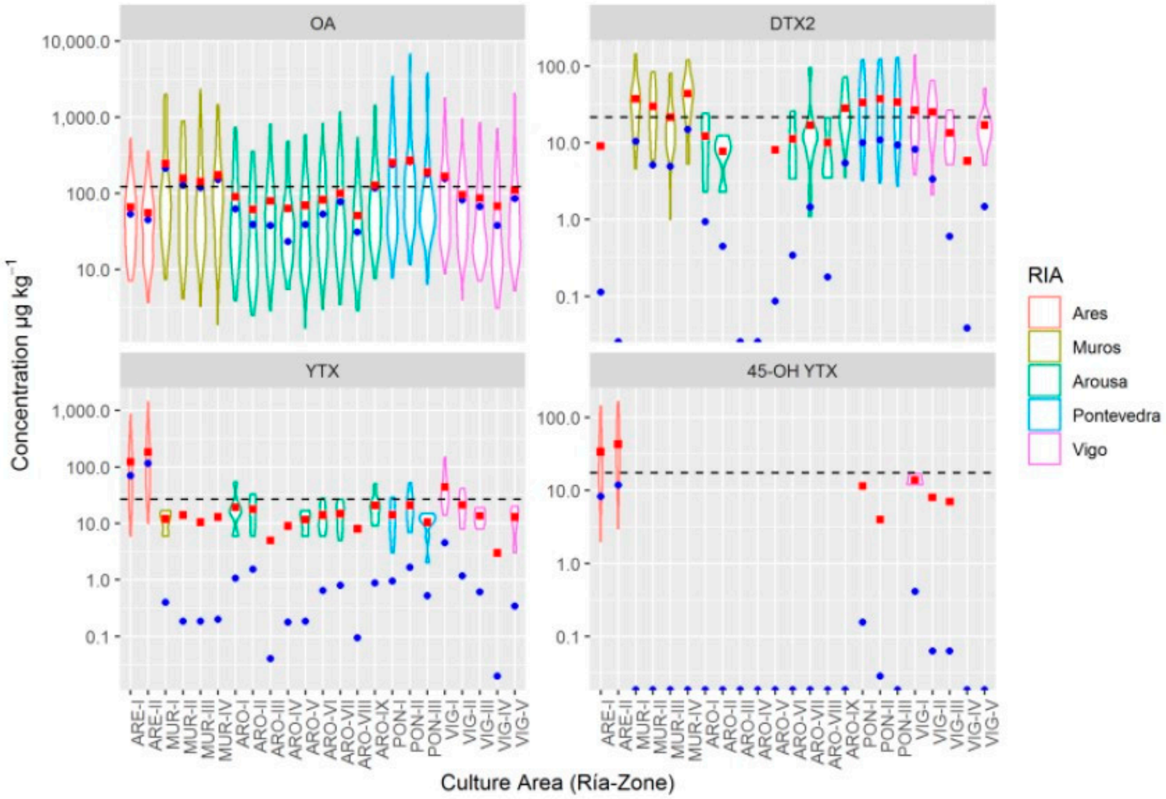

Figure S3. Violin plots of toxin concentrations in different mussel culture areas.

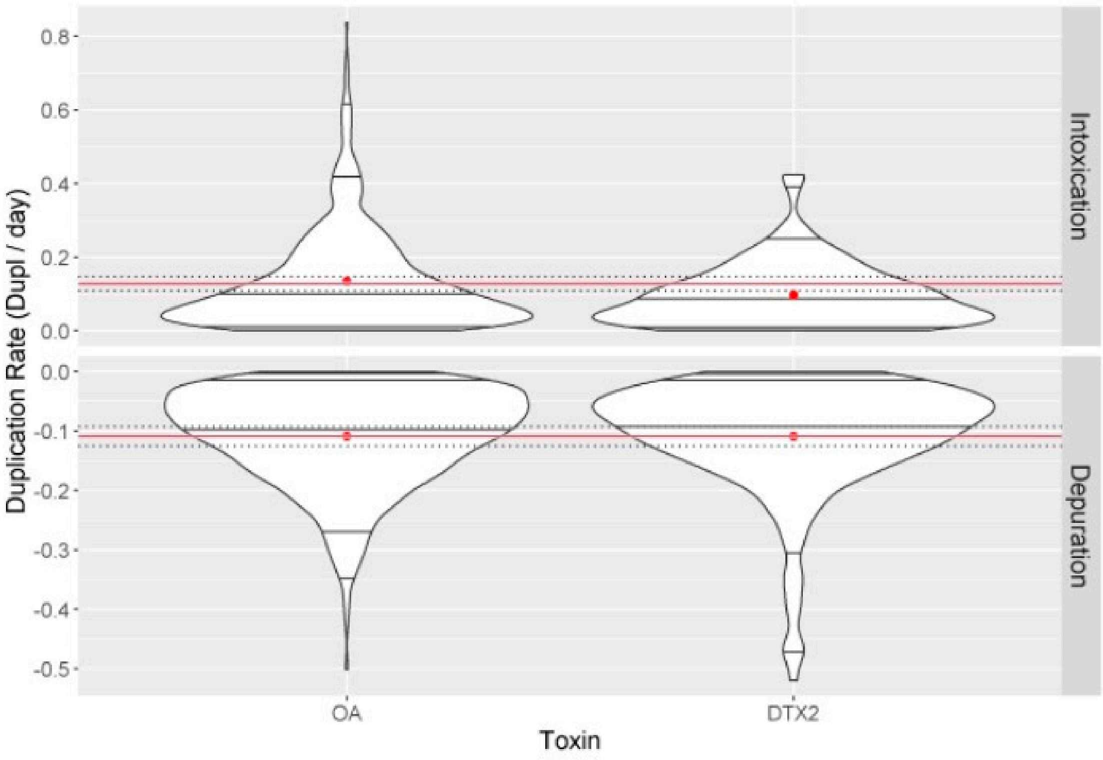

Figure S4. Violin plots of the estimated duplication rates of the toxin concentration in all the studied area.

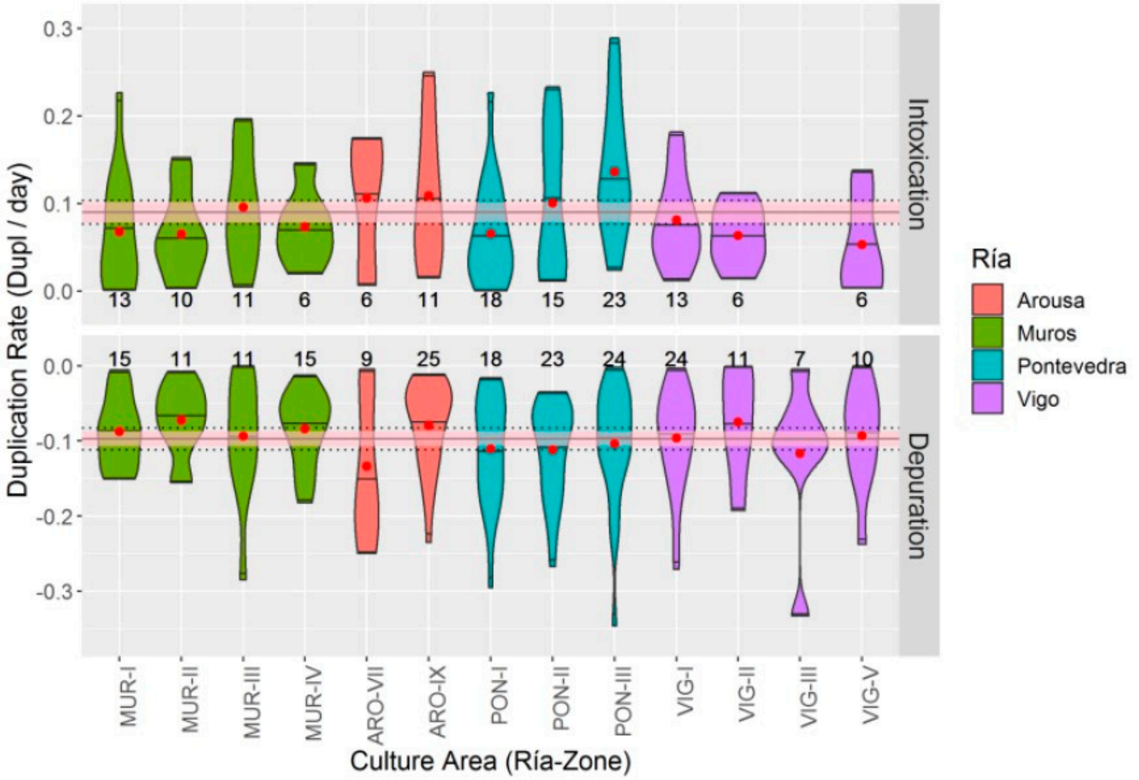

Figure S5. Plots of the duplication rates of DTX2 in the mussel culture areas after removing outliers.

Table S1. Number of samples in which all the EU regulated toxins were analysed, and numbers of samples with all toxins below and over LOD, and below and over the regulatory limit.

Species	Year	N Samples	N ≥ LOD	N < LOD	≥ Threshold	< Threshold
V. corrugata	2014	48	24	24	8	40
V. corrugata	2015	38	30	8	2	36
V. corrugata	2016	38	31	7	1	37
V. corrugata	2017	36	12	24	1	35
R. decussatus	2014	1	1	0	0	1
R. decussatus	2015	15	13	2	0	15
R. decussatus	2016	16	16	0	2	14
R. philippinarum	2014	45	30	15	2	43
R. philippinarum	2015	20	17	3	1	19
R. philippinarum	2016	45	43	2	3	42
R. philippinarum	2017	4	3	1	0	4
P. rhomboides	2014	20	20	0	5	15
P. rhomboides	2015	30	24	6	6	24
P. rhomboides	2016	19	19	0	3	16
P. rhomboides	2017	12	4	8	0	12
C. edule	2014	141	89	52	15	126
C. edule	2015	169	141	28	19	150
C. edule	2016	214	193	21	27	187
C. edule	2017	189	134	55	5	184
E. siliqua	2014	5	3	2	0	5
E. siliqua	2015	14	11	3	2	12
E. siliqua	2016	10	7	3	0	10
E. siliqua	2017	11	6	5	0	11
wild mussel	2014	79	43	36	1	78
wild mussel	2015	414	201	213	7	407
wild mussel	2016	319	248	71	14	305
wild mussel	2017	421	197	224	5	416
raft mussel	2014	389	324	65	46	343
raft mussel	2015	875	684	191	106	769
raft mussel	2016	799	740	59	193	606
raft mussel	2017	987	656	331	78	909
E. arcuatus	2014	36	28	8	6	30
E. arcuatus	2015	42	34	8	5	37
E. arcuatus	2016	12	9	3	0	12
E. arcuatus	2017	12	4	8	0	12
Ma. gigas	2016	2	2	0	0	2
O. edulis	2014	2	2	0	0	2
O. edulis	2015	4	4	0	0	4
O. edulis	2016	1	0	1	0	1
A. opercularis	2014	15	15	0	5	10
A. opercularis	2015	7	7	0	2	5
A. opercularis	2017	5	5	0	0	5
Total		5561	4074	1487	570	4991
